# A Comparative Analysis of the Effect of 24-Epibrassinolide on the Tolerance of Wheat Cultivars with Different Drought Adaptation Strategies Under Water Deficit Conditions

**DOI:** 10.3390/plants14060869

**Published:** 2025-03-10

**Authors:** Azamat Avalbaev, Ruslan Yuldashev, Anton Plotnikov, Chulpan Allagulova

**Affiliations:** Institute of Biochemistry and Genetics—Subdivision of the Ufa Federal Research Centre of the Russian Academy of Sciences, Prospect Oktyabrya 71, Lit.1e, Ufa 450054, Russia

**Keywords:** wheat (*Triticum aestivum* L.) cultivars, drought adaptation, 24-epibrassinolide, phytohormones, wheat germ agglutinin

## Abstract

Drought is a serious environmental challenge that reduces the productivity of valuable crops, including wheat. Brassinosteroids (BRs) is a group of phytohormones that have been used to enhance wheat drought tolerance. Wheat cultivars with different adaptation strategies could have their own specific drought tolerance mechanisms, and could react differently to treatment with growth regulators. In this work, the effect of seed pretreatment with 0.4 µM 24-epibrassinolide (EBR) was investigated in two wheat (*Triticum aestivum* L.) cultivars contrasting in drought behavior, tolerant Ekada 70 (cv. E70) and sensitive Zauralskaya Zhemchuzhina (cv. ZZh), in early ontogenesis under dehydration (PEG-6000) or soil drought conditions. EBR pretreatment mitigated the stress-induced inhibition of seedling emergence and growth, as well as membrane damage in cv.E70 but not in ZZh. An enzyme-linked immunosorbent assay (ELISA) revealed substantial changes in hormonal balance associated with ABA accumulation and a drop in the levels of IAA and cytokinins (CKs) in drought-subjected seedlings of both cultivars, especially ZZh. EBR-pretreatment reduced drought-induced hormone imbalance in cv. E70, while it did not have the same effect on ZZh. EBR-induced changes in the content of wheat germ agglutinin (WGA) belonging to the protective proteins in E70 seedlings suggest its contribution to EBR-dependent adaptive responses. The absence of a detectable protective effect of EBR on the ZZh cultivar may be associated with its insensitivity to pre-sowing EBR treatment.

## 1. Introduction

As a result of global climate change, there has been a significant increase in the frequency of droughts, which have a negative impact on all basic physiological processes of plants, inhibiting their growth and reducing the productivity of crops, including wheat (*Triticum aestivum* L.), which is one of the most widespread valuable cultures in the world [1,2,3,4,5,6,7]. Depending on the time of onset, spring or summer droughts are distinguished, and they can also occur throughout the entire vegetative period. Due to natural and artificial selection, wheat plants growing in various eco-geographical regions have been grouped into different ecotypes, which demonstrate different adaptation strategies to drought [8,9,10]. For example, the West Siberian forest-steppe and Volga steppe ecotypes evolved, respectively, in Western Siberia and the Volga zone, important regions for wheat production in Russia [11,12]. The differences between these ecotypes are clearly expressed at the early developmental stages, which is associated with different growing conditions. Thus, spring droughts are typical for Western Siberia; therefore, wheat cultivars of the West Siberian ecotype are characterized by slow growth and are sensitive to water stress at the initial stages of ontogenesis. In contrast, in the Volga region, drought occurs later, so cultivars of the Volga steppe ecotype grow intensively at the beginning of the growing season in order to develop an extensive root network by the time of the subsequent summer drought [11,12].

The phytohormone abscisic acid (ABA) plays an important role in plant adaptation to drought, involving the regulation of defensive programs by modulating gene expression activity encoding a wide range of RAB (responsive to ABA) proteins participating in cell protection from dehydration-induced damages [13,14]. The *Rab* family includes genes encoding wheat germ agglutinin (WGA), which is a typical representative of cereal lectins [15,16]. WGA is a constitutive protein, the content of which varies significantly in plant tissues during ontogenesis [16,17]. ABA-induced WGA accumulation was observed in wheat plants in response to various environmental stresses [18,19,20], which is accompanied by increased lectin excretion into the external environment of the roots [19,21]. The abundance of WGA in the medium surrounding the root can provide protection for stress-weakened plants from soil infection, as well as maintain the activity of the apical root meristem [20,21]. In addition to carbohydrate binding sites, wheat lectin has sites for hydrophobic interactions with non-carbohydrate molecules, which may allow for phytohormone binding, suggesting WGA’s involvement in the regulation of plant growth, development, and tissue differentiation [22]. It has previously been shown that WGA is involved in the regulation of cell division in wheat seedlings roots under water stress [20,23]. Besides ABA, other phytohormones, including brassinosteroids (BRs), are involved in the regulation of WGA synthesis in wheat seedlings [24].

Brassinosteroids are a group of steroidal phytohormones that demonstrate significant growth-promoting activity in extremely low concentrations [25]. BRs are essential for the proper regulation of a wide range of physiological processes. Moreover, a large amount of data have been accumulated that show the effectiveness of BR application in increasing the tolerance of various crops to drought, salinity, hypothermia, and other abiotic stresses [26,27,28,29]. Previously, we revealed a pronounced stimulating and protective effect of 0.4 µM 24-epibrassinolide (EBR) on the growth of wheat plants exposed to dehydration. The growth-promoting effect of EBR was accompanied by the activation of gene expression and the accumulation of WGA in wheat seedlings, suggesting that it may be involved in EBR-controlled physiological processes [16,24]. It has also been demonstrated that WGA is able to interact with EBR in the regulation of cell division in wheat roots [23]. It has been proposed that WGA may play a certain role in the protective effect of EBR on wheat seedlings exposed to unfavorable factors, including drought [16,29].

Drought is most detrimental at the initial stages of wheat ontogenesis, particularly during germination and seedling emergence, which determines all subsequent growth and eventually yield [1,2]. Thus, it is essential to uncover the physiological and biochemical changes underlying wheat drought tolerance at the early development. Various ecotypes could have their own specific mechanisms in dealing with drought conditions, especially during the early stages of the growth, and could react differently to treatment with growth regulators, such as BRs [11,12]. Thus, their effectiveness greatly depends on the cultivar’s traits, the treatment method, and the stage of plant development. Therefore, it is necessary to use an integrated approach to the study of these issues for the scientific substantiation of BR application in agricultural practice. The present study aimed to comparatively analyze the effect of pre-sowing seed treatment with 0.4 µM EBR on various physiological parameters of two wheat cultivars which belong to different eco-geographical groups: Zauralskaya Zhemchuzhina (cv. ZZh, West Siberian forest-steppe ecotype) and Ekada 70 (cv. E70, Volga steppe ecotype) at the initial stages of growth under drought conditions, modulated by polyethylene glycol (PEG-6000) or limited irrigation. The differences in the intensity of germination, biomass accumulation, changes in hormonal balance, WGA accumulation, and membrane permeability was investigated in wheat seedlings of both cultivars pretreated with EBR and exposed to drought stress.

## 2. Results

### 2.1. Effect of 24-Epibrassinolide Pretreatment on Seed Germination and Early Growth of Wheat Seedlings Under PEG Exposure

**Figure 1:**
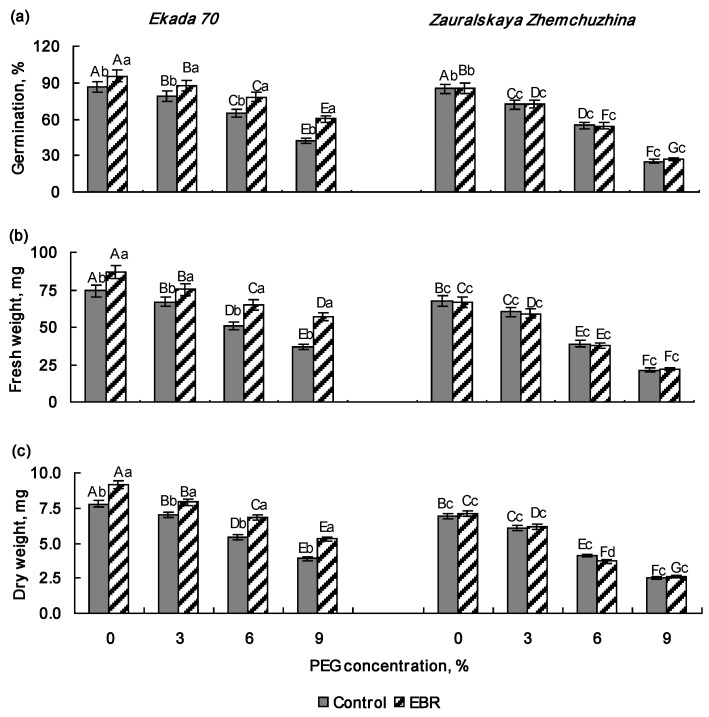
The effect of pre-sowing seed treatment with 0.4 µM 24-epibrassinolide on seed germination (**a**) and fresh (**b**) and dry weights (**c**) of 5-day-old whole seedlings of the Ekada 70 and Zauralskaya Zhemchuzhina cultivars under drought conditions induced by PEG-6000 solutions. Mean values and their standard errors are presented (*n* = 3). Different lowercase letters on top of the columns indicate that means for the same time point of various treatments are different at *p* < 0.05 (analysis of variance (ANOVA), least significant difference (LSD) test). Different capital letters on top of the columns indicate that means for each treatment at different time points are different at *p* < 0.05 (ANOVA, LSD test).

Initially, the effect of 0.4 µM 24-epibrassinolide on seed germination and early wheat growth was evaluated by the emergence and changes in fresh and dry weights of 5-day-old whole seedlings of the Ekada 70 and Zauralskaya Zhemchuzhina cultivars under normal and drought conditions induced by PEG-6000 solutions in concentrations of 0%, 3%, 6%, and 9% (Figure 1).

The E70 cultivar had a better germination percentage and growth indices than ZZh. Pre-sowing EBR treatment increased seed germination by about 11% (Figure 1a), and the accumulation of fresh and dry weight by 17% and 18%, respectively, in 5-day-old seedlings of the E70 cultivar (Figure 1b,c), but had no significant growth-promoting effect in the ZZh cultivar as compared to the control EBR-untreated samples (Figure 1a–c). Exposure to drought inhibited seed germination and biomass accumulation in the both wheat ecotypes, and their response was dependent on PEG concentrations. The negative effect of PEG was already noticeable at a concentration of 3%, and the decrease in all studied parameters varied within the range of 7–9% in cv. E70 and 11–15% in cv. ZZh. As the stress level increased, the negative effects became more pronounced. The presence of 6% PEG in the growth medium reduced seed germination by about 24% and 36% in cultivars E70 and ZZh, respectively, and also accumulation of the fresh and dry weight in the 5-day-old seedlings by about 32% and 30% in cv. E70 and by 43% and 41% in cv. ZZh, respectively. An increase in the PEG concentration to 9% reduced all studied growth parameters by nearly 50% and 70% in cv. E70 and cv. ZZh, respectively. These data indicate a higher sensitivity of the ZZh cultivar to water deficits at the early stages of growth. Pre-sowing EBR treatment contributed to prevention of growth inhibition caused by 3% PEG and significantly mitigated the harmful effect of 6% and 9% PEG on 5-day-old E70 seedlings. Meanwhile, no remarkable improvements were detected in the growth indicators of PEG-exposed ZZh seedlings due to EBR pretreatment.

### 2.2. The Effect of 24-Epibrassinolide Seed Pretreatment on Growth of Wheat Seedlings Exposed to Soil Drought in Pot Experiments

**Figure 2:**
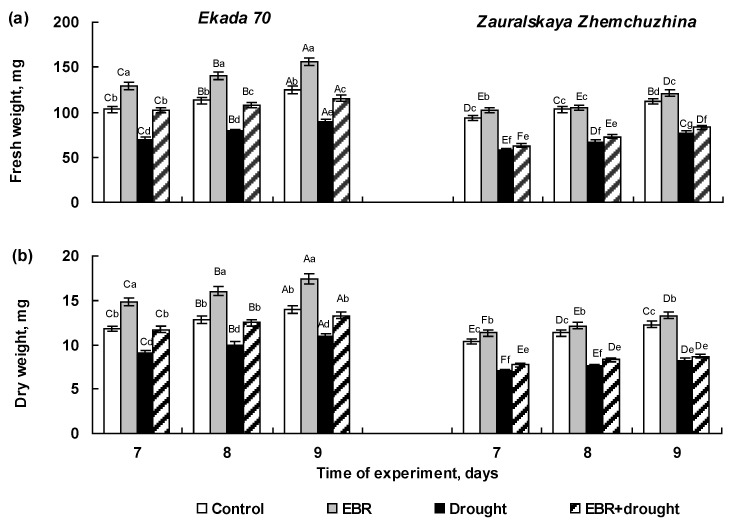
The effect of pre-sowing seed treatment with 0.4 µM 24-epibrassinolide on fresh (**a**) and dry weights (**b**) of 7-, 8-, 9-day-old whole seedlings of the Ekada 70 and Zauralskaya Zhemchuzhina cultivars under soil drought conditions. Mean values and their standard errors are presented (*n* = 3). Different lowercase letters on top of the columns indicate that means for the same time point of various treatments are different at *p* < 0.05 (ANOVA, LSD test). Different capital letters on top of the columns indicate that the means for each treatment at different time points are different at *p* < 0.05 (ANOVA, LSD test).

The intensity of wheat growth in the pot experiments was evaluated by the dynamics of the fresh and dry weight of the 7-, 8-, and 9-day-old whole seedlings of both cultivars pretreated and untreated with 0.4 µM EBR and subjected to soil drought, which was modeled by limiting irrigation to a relative soil moisture content at 30% of the maximal soil water-holding capacity. Control seedlings of cv. E70 had higher values of fresh and dry weights compared to the cv. ZZh (Figure 2a,b). The biomass values of E70 seedlings exceeded those of ZZh by about 10–12%. Soil drought led to a noticeable inhibition of the growth indices in the both cultivars, but the decline in the fresh (Figure 2a) and dry (Figure 2b) weights was more substantial in ZZh plants. The stress-induced reduction in the fresh and dry weights of 7–9-day-old E70 seedlings were approximately 30% and 21%, while in ZZh seedlings, it reached 40% and 34%, respectively (Figure 2a,b). These results indicate a higher sensitivity of ZZh plants to soil drought at the early stage of growth.

Pre-sowing EBR-treatment had a clear growth-promoting effect on the seedlings of cv. Ekada 70 under normal conditions stimulating an increase in biomass accumulation by about 25%, whereas the fresh and dry weights of EBR-pretreated seedlings of cv. Zauralskaya Zhemchuzhina remained approximately at the control levels (Figure 2). It was revealed that EBR significantly decreased the negative effect of soil drought on the growth of E70 seedlings. The fresh and dry weight of EBR-pretreated seedlings and those subjected to stress E70 plants were comparable to those of the control. At the same time, pre-sowing EBR treatment had no a protective effect on the growth of ZZh seedlings exposed to drought. Their fresh and dry weights were not significantly different from the values of samples untreated with EBR and subjected to stress (Figure 2). Thus, the E70 cultivar exhibited better drought tolerance during the initial stages of development compared to the ZZh cultivar. EBR seed pretreatment had a protective effect against soil drought on the early growth of wheat plants of cv. E70, but not of cv. ZZh.

### 2.3. The Effect of 24-Epibrassinolide Seed Pretreatment on Hormonal Balance of Wheat Seedlings Subjected to Soil Drought

**Figure 3:**
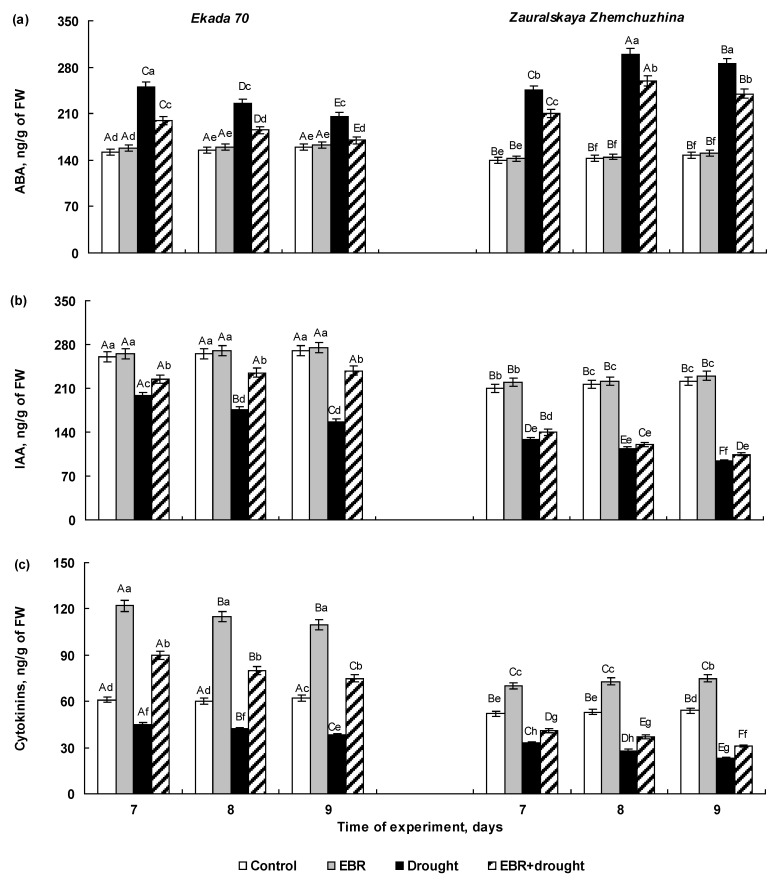
The effect of pre-sowing seed treatment with 0.4 µM 24-epibrassinolide on the contents of ABA (**a**), IAA (**b**), and cytokinins (**c**) in 7-, 8-, and 9-day-old seedlings of the Ekada 70 and Zauralskaya Zhemchuzhina cultivars under soil drought conditions. Mean values and their standard errors are presented (*n* = 3). Different lowercase letters on top of the columns indicate that means for the same time point of various treatments are different at *p* < 0.05 (ANOVA, LSD test). Different capital letters on top of the columns indicate that means for each treatment at different time points are different at *p* < 0.05 (ANOVA, LSD test).

The results of an enzyme-linked immunosorbent assay (ELISA) of abscisic acid (ABA), indolylacetic acid (IAA), and cytokinins (CKs) in EBR-pretreated 7-, 8-, and 9-day-old wheat seedlings of two cultivars with contrasting drought behavior under normal and soil drought conditions are presented in Figure 3.

The control plants of cv. E70 differed from cv. ZZh by a higher concentration of IAA and CKs, but not of the ABA (Figure 3). The pre-sowing EBR-treatment had no significant effect on the content of ABA (Figure 3a) and IAA (Figure 3b) in both cultivars; however, it induced more than a 2-fold accumulation of cytokinins in E70 plants (Figure 3c). EBR-pretreated seedlings of cv. ZZh were also characterized by an increased level of cytokinins, but it was significantly lower compared to the E70 plants. The cytokinins content in EBR-pretreated 7–9-day-old ZZh seedlings was higher than in EBR-untreated ones by approximately 35–40% according to the immunoassay (Figure 3c).

Drought stress induced considerable changes in the hormonal status of both cultivars, associated with a rapid accumulation of ABA and a decrease in the concentrations of the growth-stimulating hormones IAA and CKs (Figure 3a–c). In the E70 plants, the greatest drought-induced increase in ABA content, amounting about 64% from the control level, was detected in the 7-day-old seedlings. In 8- and 9-day-old E70 plants, the increase in ABA content caused by stress was less significant and exceeded the control by about 45% and 30%, respectively (Figure 3a). A decrease in the content of IAA by 23%, 34%, and 41%, and of cytokinins by 26%, 30%, and 38%, in comparison to the control was detected in E70 seedlings on the 7th, 8th, and 9th day of the experiment, respectively (Figure 3b,c). The accumulation of ABA in the 7-day-old ZZh seedlings increased by 75% relatively, then by the 8th day its content increased more than 2-fold and remained at almost the twice higher level than in control in the 9-day-old plants (Figure 3a). Stress caused a decrease in the IAA content in the ZZh seedlings by 39%, 45%, and 55%, and cytokinins by 37%, 47%, and 58% on the 7th, 8th, and 9th days of the experiment, respectively (Figure 3b,c). Therefore, the stress-induced changes in hormonal balance were more dramatic in the cultivar Zauralskaya Zhemchuzhina (Figure 3), which eventually leads to a stronger growth inhibition in comparison to the cultivar Ekada 70 (Figure 2).

The EBR application contributed to the normalization of the phytohormonal balance in the seedlings of the cv. E70 subjected to drought. This was evident from the decrease in stress-induced ABA accumulation with a simultaneous increase in IAA and CK content, compared to the EBR-untreated and drought-exposed samples (Figure 3a–c). Moreover, even under stressful conditions, EBR-pretreated Ekada 70 seedlings were characterized by elevated content of CKs compared to the control throughout the whole experiment (Figure 3c). Remarkably, in EBR-pretreated Zauralskaya Zhemchuzhina plants, stress-induced accumulation of ABA was at a significantly higher level relative to the control (Figure 3a). The content of auxins and cytokinins in these plants were identical to those of EBR-untreated and drought-exposed plants (Figure 3b,c). This was reflected in the absence of a distinguishable positive effect of EBR-pretreatment on the growth indices of seedlings of the Zauralskaya Zhemchuzhina cultivar under both normal and stressful conditions (Figure 2).

### 2.4. The Effect of 24-Epibrassinolide Seed Pretreatment on WGA Accumulation of Wheat Seedlings Subjected to Soil Drought

**Figure 4:**
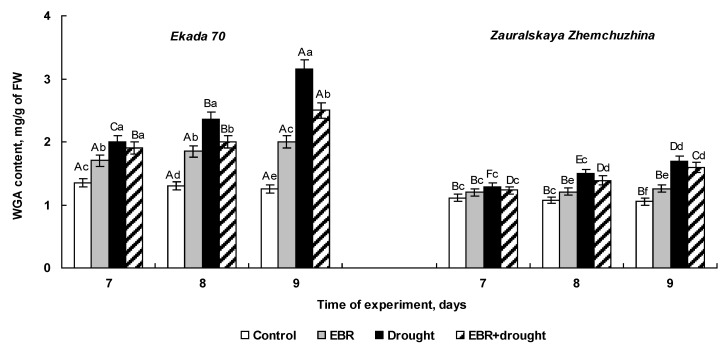
The effect of pre-sowing seed treatment with 0.4 µM 24-epibrassinolide on WGA content in 7-, 8-, and 9-day-old seedlings of the Ekada 70 and Zauralskaya Zhemchuzhina cultivars under soil drought conditions. Mean values and their standard errors are presented (*n* = 3). Different lowercase letters on top of the columns indicate that means for the same time point of various treatments are different at *p* < 0.05 (ANOVA, LSD test). Different capital letters on top of the columns indicate that means for each treatment at different time points are different at *p* < 0.05 (ANOVA, LSD test).

We compared the dynamics of quantitative changes in WGA content in 7-, 8-, and 9-day-old seedlings of both varieties untreated and pretreated with EBR and exposed to soil drought. The level of wheat lectin in control seedlings was stable throughout the whole experiment in both cultivars, although the cv. Ekada 70 was characterized by a somewhat higher level of WGA in comparison with the Zauralskaya Zhemchuzhina seedlings (Figure 4).

Under normal conditions, pre-sowing seed treatment with EBR caused the 25% increase in WGA content in Ekada 70 seedlings. However, no changes in the content of wheat lectin were revealed in Zauralskaya Zhemchuzhina plants (Figure 4).

Drought stress resulted in a gradual increase in WGA content in wheat seedlings while the dynamics of lectin accumulation differed between the studied cultivars. Drought-tolerant cv. E70 was distinguished by a higher level of stress-induced WGA accumulation in comparison to cv. ZZh. The WGA content increased almost 1.5-fold in 7-day-old Ekada 70 seedlings in response to drought reaching a maximum level that was 250% of the control on the 9th day of the experiment. A noticeable accumulation of wheat lectin in Zauralskaya Zhemchuzhina plants was registered only on the 9th day, reaching 150% of the control level.

Under soil drought conditions, EBR-pretreated Ekada 70 plants were characterized by a lower level of WGA accumulation compared to hormone-untreated seedlings (Figure 4). At the same time, there was no significant effect of EBR treatment on WGA content in Zauralskaya Zhemchuzhina seedlings under stress conditions. Thus, we have identified substantial differences between two wheat cultivars differing in drought-tolerance in terms of WGA content, as well as in the dynamics of the accumulation of this protein in wheat seedlings of both investigated cultivars pretreated with EBR under normal and soil drought conditions.

### 2.5. The Effect of 24-Epibrassinolide Seed Pretreatment on Drought-Induced MDA Accumulation and Elektrolyte Leackage in Wheat Seedlings

Drought increased malondialdehyde (MDA) production and the level of electrolyte leakage in E70 seedlings by about 70% and 75%, respectively, whereas both of these parameters were increased approximately 2-fold in ZZh plants in comparison to the control at the 9th day of the experiment (Table 1).

These results indicate that the drought-induced membrane damage was more severe in Zauralskaya Zhemchuzhina plants compared to Ekada 70. EBR seed pretreatment of cv. E70 reduced stress-evoked MDA accumulation and electrolyte leakage by about 25% and 30%, respectively, in 9-day-old seedlings, demonstrating the protection of the membrane structures’ integrity. EBR pretreatment had no effect on the stress-induced MDA production and electrolyte leakage level in ZZh seedlings, which indicates the absence of a protective effect of pre-sowing EBR treatment on membrane structures in cv. Zauralskaya Zhemchuzhina.

## 3. Discussion

Drought is one of the most common abiotic environmental stresses which significantly reduce the growth and metabolism of valuable crops, including wheat, especially at the early stages of development [7,30,31]. During this period, wheat seeds and seedlings are characterized by high sensitivity to water stress [2,32]. Thus, we expected the observed inhibition of seed germination and the growth of 5-day-old seedlings in both investigated wheat cultivars (E70 and ZZh) in response to PEG exposure (Figure 1 and Figure 2). At the same time, the cv. E70 seedlings were characterized by higher growth indices under drought modulated by a PEG gradient in comparison to cv. ZZh (Figure 1a–c). Further, our results from pot experiments demonstrate that 7–9-day-old plants of cv. E70 also maintain higher growth parameters in comparison with the ZZh plants under soil drought conditions (Figure 2a,b). This is in accordance with the fact that wheat plants belonging to the Volga steppe ecotype (cv. E70) have better drought tolerance compared to those of the West Siberian ecotype (cv. ZZh) at the early stages of development [12].

Brassinosteroids (BRs) are an important group of phytohormones, and their role in plant responses to abiotic stress is becoming increasingly evident. A considerable amount of data have been accumulated to date on the effectiveness of BR use in enhancing wheat tolerance to water stress [27,28,33]. In this study, it was found that EBR pre-sowing treatment improved seed germination and growth rates of wheat seedlings belonging to the cultivar E70, both under normal and drought conditions (Figure 1 and Figure 2). Similar results were also reported by numerous studies showing that brassinosteroids could mitigate the harmful effect of drought stress on the growth of different plant species, including wheat [34,35,36,37]. However, in the case of the cv. ZZh, EBR had no visible protective effect on seed germination and seedling growth under drought stress (Figure 1 and Figure 2). Thus, EBR pretreatment increased the tolerance of cv. E70 wheat seedlings to drought, whereas hormone application did not enhance protection against water stress in ZZh plants (Figure 1 and Figure 2). The difference in the response of cultivars to EBR treatment under drought conditions may be associated with their distinct drought adaptation strategies. Wheat plants of ZZh cultivar (West Siberian forest-steppe ecotype) are distinguished by slow growth and development in early ontogenesis, which enables them to withstand spring drought [9,12]. Therefore, the application of EBR did not promote the growth of ZZh plants, since rapid and intensive growth is not determined by their development program during the initial stage of ontogenesis. In contrast, the E70 plants (Volga steppe ecotype) “programmed” for intensive growth at the beginning of the vegetation period improved growth after hormone pretreatment under normal and drought stress [9,10,12]. Understanding the mechanisms underlying the interactions of brassinosteroids and wheat cultivars differing in their drought tolerance-adaptive strategies is important to improve the approaches for the using of plant growth regulators in agriculture.

It is known that plant hormones play a leading role in the regulation of physiological processes at all stages of ontogenesis, including at the young seedling stage [38,39,40]. Thus, the effect of EBR pretreatment on the growth of the studied wheat cultivars may be determined by its effect on the endogenous hormonal system. As expected, typical stress-related changes in hormonal balance [38,41] were observed in plants of both ecotypes under water stress—a considerable accumulation of ABA and a significant drop in levels of IAA and cytokinins; however, these changes were more pronounced in seedlings of cv. ZZh. Previously, it was found that wheat genotypes with contrasting drought tolerance were distinguished by different levels of stress-induced endogenous ABA [42]. Thus, drought stress enhanced the expression of genes involved in ABA biosynthesis and promoted ABA accumulation in drought-sensitive wheat cultivars. In contrast, drought-tolerant wheat cultivars were characterized by lower ABA levels, which correlated with a slower biosynthesis of ABA and a higher level of gene expression associated with ABA-catabolic pathways [42]. In accordance with these data, it was found in our work that drought stress resulted in a significant increase in ABA content in the seedlings of drought-sensitive cv. ZZh compared to the drought-tolerant cv. E70 (Figure 3a). Additionally, there was a persistent decrease in the levels of IAA and CK (Figure 3b,c), which led to the inhibition of growth rate in ZZh plants (Figure 2).

Seed priming with EBR reduced stress-induced fluctuations in the balance of plant hormones in cv. E70 seedlings. Thus, EBR-pretreated E70 plants were characterized by lower drought-induced ABA accumulation and IAA decrease in comparison with EBR-untreated ones. It should be noted that EBR pretreatment not only prevented a decline in the content of cytokinins, but also maintained elevated levels of these phytohormones which are known by their pronounced antistress action [43,44,45]. Indeed, EBR-pretreated drought-stressed E70 plants were distinguished by improved growth than that of EBR-untreated stressed seedlings. At the same time, in drought-stressed plants of cv. ZZh, EBR did not reduce stress-induced shifts in the hormonal system (Figure 3), which was reflected in the lack of protective effect of EBR on seedling growth (Figure 2).

Exposure of plants to drought enhanced the accumulation of a wide range of stress proteins, including wheat germ agglutinin [18,19,46]. A large body of data has been obtained that demonstrate a significant increase in the content of lectin in wheat plants exposed to abiotic stress factors causing dehydration in plant tissues, such as salinity [29], hyperthermia [46], heavy metals [20,21], drought, and osmotic stress [16,18], suggesting WGA involvement in the protective reactions of wheat in response to dehydration. The data obtained in this study strongly indicate that wheat cultivars with contrasting drought tolerance were distinguished by a different content of WGA at early ontogeny (Figure 4). Thus, E70 plants, tolerant to early drought, were characterized by a higher level of stress-induced WGA accumulation than those of the ZZh cultivar (Figure 4). The ability of EBR to stimulate additional lectin synthesis under normal conditions and maintain its content in Ekada 70 plants exposed to drought at the level close to those of unstressed samples may indicate a significant contribution of WGA to the protective effect of EBR on E70 cultivar to drought. Since WGA is a protein excreted into the external environment, it can be assumed that a stress-induced increase in its level leads to increased excretion of lectin into the environment surrounding the roots, which may play an important role in protecting the rhizosphere from infection by phytopathogens. This is likely due to the affinity of lectin for N-acetyl-D-glucosamine, a chitin monomer, an important component of the cell walls of chitin-containing pathogenic fungi [15,29]. Among proposed functions of WGA, its role in the regulation of cell division is considered, which also suggest the involvement of WGA in the maintenance of the proliferation of meristematic cells in seedlings exposed to stress [16,22]. This may be supported by the data on promotive combined effect of EBR and WGA treatment on cell division in wheat root apices [23]. Moreover, the application of EBR was found to maintain cell division in wheat seedling roots under stress conditions, and EBR-induced accumulation of WGA may contribute to this effect [16].

Drought has been known to induce a rapid rise in ROS production, causing damage to membrane integrity and an increase in membrane permeability [47,48]. As a result, drought-induced oxidative stress provokes a considerable accumulation of malondialdehyde and an increase in the level of electrolyte leakage, which are reliable indicators of membrane damage [27,32]. Indeed, under soil drought conditions, wheat seedlings of both ecotypes were distinguished by an increase in MDA levels and electrolyte leakage (Table 1). Stressed seedlings of the ZZh cultivar had higher levels of MDA and electrolyte leakage than that of the E70 cultivar (Table 1), which confirmed the lower drought tolerance of ZZh plants. Numerous studies have reported that BRs could reduce stress-induced MDA accumulation and electrolyte leakage by activating a strong ROS scavenging system [49,50]. EBR did not improve the integrity of membrane structures and drought tolerance in plants of the ZZh cultivar, as EBR-pretreated ZZh seedlings had almost identical MDA and electrolyte leakage values as hormone-untreated ones. However, EBR pretreatment diminished the drought-induced increase in these parameters in Ekada 70 plants, contributing to the development of effective antistress protection in this cultivar (Table 1).

The obtained results convincingly indicate the effectiveness of pre-sowing seed treatment with 0.4 µM EBR of Ekada 70 plants of the Volga steppe ecotype in improving their drought tolerance at the initial stage of ontogeny. Indeed, the present research has demonstrated that EBR-pretreated drought-stressed E70 seedlings experience less damage than hormone-untreated plants, as evidenced by improved seed germination and growth indices, as well as lower MDA accumulation and leakage of electrolytes (Figure 1 and Figure 2; Table 1). EBR-induced drought tolerance in E70 seedlings is based on the normalization of rapid stress-induced changes in the level of endogenous phytohormones. The ability of EBR to modulate the accumulation of wheat germ agglutinin, an important component of the defensive wheat programs, may contribute to the development of EBR-induced drought protective reactions in plants of the Ekada 70 cultivar. At the same time, the lack of a positive effect of 24-epibrassinolide on plants of the Zauralskaya Zhemchuzhina cultivar belonging to the West Siberian forest-steppe ecotype may be associated with their insensitivity to pre-sowing EBR treatment.

## 4. Materials and Methods

### 4.1. Seed Material, Pre-Sowing EBR-Treatment

Seeds of soft wheat *Triticum aestivum* L., of two cultivars differing in drought behavior, drought-tolerant Ekada 70 (cv.E-70) and drought-sensitive Zauralskaya Zhemchuzhina, were obtained from the Chishminsky Breeding Centre (Republic of Bashkortostan, Russia). The seeds were sterilized by immersing them in 96% ethanol for 60 s, then they were washed in running water and blotted with filter paper to remove the excess moisture. The seeds were soaked for 3 h in a solution of 24-epibrassinolide at a concentration of 0.4 µm which, as was previously shown, is effective in the stimulation and protection of wheat seedling growth [24,29]. Control seeds were soaked in distilled water.

### 4.2. Assessment of Seed Germination and Early Seedling Growth Assays

EBR-pretreated and untreated seeds were sown in Petri dishes covered with filter paper drenched with PEG-6000 solutions at concentrations of 0%, 3%, 6%, and 9%. The seeds were germinated in a thermostat TS-1/80 SPU (Smolensk, Russia) at 21–22 °C in the dark. The percentage ratio of emerging seedlings to the total number of sown seeds was counted after 5 days of germination. Early seedling growth was estimated by measuring the differences in fresh and dry weights of 5-day-old seedlings.

### 4.3. Pot Experiment Design, Treatments, and Soil Drought Conditions

Pretreated and untreated with EBR seeds were grown on filter paper with tap water for 3 days under a specific photoperiod (16 h light/8 h dark, 21–22 °C). Then, 3-day-old seedlings were transferred to pots (25 × 25 × 25 cm) with soil (optimal NPK ratio, pH 6.5, humidity 60%) (Veltorf LLC, Velikiye Luki, Russia). The distance between the plants in the rows and between the rows was 3 cm. Agrotechnical clay (10–20 mm) was preliminary placed on the bottom of each pot (Florizel, Terra Master Ltd., Novosibirsk, Russia) and then they were filled with soil. After planting, wheat seedlings were grown under controlled conditions (16/8 h day/night photoperiod, 21–22 °C, 60% relative air humidity). Experimental plants were divided into 2 groups: (1) those grown under normal irrigation at optimal soil moisture contents (70% of the maximal soil water-holding capacity); and (2) those exposed to soil drought, which was modeled by limiting irrigation to a relative soil moisture content at 30% of the maximal soil water-holding capacity. Soil moisture content was monitored gravimetrically [51]. Samples were collected at 7, 8, and 9 days of germination, fixed in liquid nitrogen, and stored at −70 °C for further evaluation of physiological and biochemical parameters. Fresh weight was measured immediately after harvesting. To determine the dry weight, the plant material was air-dried at 80 °C until a constant weight was achieved. Each variant was carried out in three replicates, with 25 plants per replicate.

### 4.4. Immunoassay of Phytohormones and WGA

The contents of abscisic acid (ABA), indoleacetic acid (IAA), cytokinins (CKs), and wheat germ agglutinin (WGA) were measured in 10 wheat seedlings (1–1.5 g of fresh weight) by enzyme-linked immunosorbent assay (ELISA) using specific polyclonal rabbit antibodies against each of the investigated compound and anti-rabbit antibodies labeled with peroxidase as described earlier [23]. Plant material was homogenized in 80% ethanol (1:10, *w*/*v*) and kept at 4 °C for 16 h to extract phytohormones, while WGA remained insoluble in the pellets. CKs were immunoassayed in aqueous residues of ethanol extracts. We have determined the total content of zeatin derivatives (zeatin, its riboside, and nucleotide) immunoreactive in the test system with rabbit antibodies to zeatin riboside [23]. For ABA and IAA measurement, the remaining aqueous residue was acidified with HCl to pH 2.5 and partitioned twice with diethyl ether. After that, ABA and IAA were transferred from the organic phase into 1% sodium hydrocarbonate (pH 7–8), re-extracted with diethyl ether, methylated with diazomethane, and immunoassayed using polyclonal antibodies to ABA and IAA [23]. WGA was extracted with 0.05 M HCl for 1 h at room temperature from the pellets remaining after phytohormone extraction. The supernatants were neutralized with 1MNa–phosphate buffer (pH 7.2) and then were used for WGA estimation by ELISA [23,31].

### 4.5. Measurements of MDA and Electrolyte Leakage

The content of malondialdehyde (MDA), one of the main products of lipid peroxidation, was determined as described in [52]. About 1 g of seedling tissue was homogenized in 10% trichloroacetic acid (TCA), and the homogenate was centrifuged at 12,000× *g* for 20 min. The supernatant was mixed with an equal volume of thiobarbituric acid (0.5% in 20% [*w*/*v*] TCA), and the mixture was boiled for 30 min at 100 C, followed by centrifugation at 8000× *g* for 15 min. After centrifugation, the optical density of the supernatant was recorded at 532 nm. The membrane permeability of wheat seedling roots was evaluated by measuring electrolyte leakage from the plant tissues [52]. Seedling samples (about 1 g) were washed with tap water, cut, and incubated in distilled water at 27 °C for 1 h. After filtration, the electrolyte leakage of the water extract was measured with the conductivity meter HI8733 (Hanna Instruments, Inc., Woonsocket, RI, USA).

### 4.6. Statistical Analysis

All experiments were performed at least three times. The experimental data were subjected to a one-way analysis of variance (ANOVA) using SPSS 13.0 software for Windows (SPSS Inc., Chicago, IL, USA). The significant differences between mean values were estimated using the least significant difference (LSD) test at *p* < 0.05. The data in the figures and table are presented as mean values and their standard errors (±SE).

## 5. Conclusions

Drought is one of the most widespread and unpredictable environmental stresses that pose a serious challenge for the production of agricultural crops, including valuable cultures such as wheat. Drought is especially destructive at the initial stages of ontogenesis, such as seed germination and seedling emergence, because they determine all subsequent growth and development and, ultimately, the plant’s productivity. Various agrochemicals are widely used in practice to reduce crop losses. However, such compounds can pose a serious threat to the environment and human health when applied in excessive amounts. A promising approach to improve plant productivity and stress tolerance is the application of nature growth regulators, in particular, brassinosteroids (BRs). Numerous studies have demonstrated their role not only in the regulation of normal plant development, but also in enhancing drought tolerance in various plant species, including wheat. The effectiveness of BR application may vary significantly depending on the type of treatment, developmental stage, and cultivar traits. Moreover, various ecotypes which are characterized by specific drought tolerance mechanisms could react differently to treatment with BRs. In this work, we conducted a comparative analysis of the effect of pre-sowing treatment with 0.4 µM 24-epibrassinolide (EBR) on the growth, hormonal balance, accumulation of WGA protein, and maintenance of membrane structures in two wheat cultivars belonging to ecotypes with different drought adaptation strategies contrasting in drought tolerance: Zauralskaya Zhemchuzhina (sensitive cultivar) and Ekada 70 (tolerant cultivar) at the initial stages of the growth under water stress conditions. The obtained results clearly demonstrate the differences in the response of the studied cultivars to EBR-pretreatment under normal and water stress conditions. Pre-sowing EBR treatment contributed to growth activation at the initial stages of ontogenesis in cv. E70, both under normal and drought conditions, and had no significant effect on the growth of ZZh seedlings. Such discrepancies can be explained by different drought adaptation strategies for studied cultures. Wheat plants of the ZZh variety are cultivated in the West Siberian forest-steppe region, in which spring droughts often occur. Therefore, rapid growth during early ontogenesis is not beneficial for the survival of ZZh plants in conditions of spring drought, which may explain the absence of EBR-promoting effect on their growth. At the same time, wheat plants of the E70 cultivar are grown mainly in the Volga steppe region, which is characterized by summer droughts. These plants are “programmed” for intensive growth at the beginning of the vegetation period, determining the formation of a well-developed root system, which is essential for improved survival in summer drought conditions. Thus, the EBR-induced activation of growth processes at the initial stages of the development of E70 plants can additionally increase their resistance to the subsequent impact of drought. The total experimental data obtained in this work suggests that the growth-promoting and protective effects of EBR pretreatment on E70 plants may be due to its influence on their hormonal system, regulation of WGA protein synthesis, and stabilization of their membrane structures.

## Figures and Tables

**Table 1:** The effect of 0.4 μM EBR seed pretreatment on the content of malondialdehyde (MDA) and the level of electrolyte leakage (EL) in 9-day-old wheat seedlings of the cultivars Ekada 70 (E70) and Zauralskaya Zhemchuzhina (ZZh) subjected to soil drought.

Treatment	MDA, mol g^−1^ Fresh Weight		EL, µS/g Fresh Weight	
	E70	ZZh	E70	ZZh
Control	41.3 ± 2.1 Cb	49.9 ± 2.5 Ba	20.1 ± 0.9 Cb	23.3 ± 1.1 Ba
EBR	43.8 ± 2.2 Cb	51.7 ± 2.6 Ba	21.2 ± 1.0 Cb	24.4 ± 1.2 Ba
Drought	70.3 ± 3.5 Ab	98.3 ± 4.1 Aa	35.1 ± 1.7 Ab	47.8 ± 2.3 Aa
EBR + drought	52.4 ± 2.6 Bb	91.7 ± 3.8 Aa	25.7 ± 1.2 Bb	43.5 ± 2.1 Aa

The data shown are the mean values and their standard errors (*n* = 3). Different lowercase letters indicate that means in the same row for the same parameter are significantly different at *p* < 0.05 (ANOVA, LSD test). Different capital letters indicate that means in the same column are significantly different at *p* < 0.05 (ANOVA, LSD test).

## Data Availability

The data are contained within the article.
